# Insights about the process and impact of implementing nursing guidelines on delivery of care in hospitals and community settings

**DOI:** 10.1186/1472-6963-8-29

**Published:** 2008-02-02

**Authors:** Barbara Davies, Nancy Edwards, Jenny Ploeg, Tazim Virani

**Affiliations:** 1 School of Nursing, Faculty of Health Sciences, University of Ottawa, Ottawa, Canada; 2 Department of Epidemiology and Community Medicine, Faculty of Medicine, University of Ottawa, Canada; 3 School of Nursing, Faculty of Health Sciences, McMaster University, Hamilton, Canada; 4 Department of Health Policy, Management and Evaluation, Faculty of Medicine, University of Toronto, Toronto, Canada; 5 Tazim Virani & Associates, Health & Social Services Research and Management Consultants, Canada

## Abstract

**Background:**

Little is known about the impact of implementing nursing-oriented best practice guidelines on the delivery of patient care in either hospital or community settings.

**Methods:**

A naturalistic study with a prospective, before and after design documented the implementation of six newly developed nursing best practice guidelines (asthma, breastfeeding, delirium-dementia-depression (DDD), foot complications in diabetes, smoking cessation and venous leg ulcers). Eleven health care organisations were selected for a one-year project. At each site, clinical resource nurses (CRNs) worked with managers and a multidisciplinary steering committee to conduct an environmental scan and develop an action plan of activities (i.e. education sessions, policy review). Process and patient outcomes were assessed by chart audit (n = 681 pre-implementation, 592 post-implementation). Outcomes were also assessed for four of six topics by in-hospital/home interviews (n = 261 pre-implementation, 232 post-implementation) and follow-up telephone interviews (n = 152 pre, 121 post). Interviews were conducted with 83/95 (87%) CRN's, nurses and administrators to describe recommendations selected, strategies used and participants' perceived facilitators and barriers to guideline implementation.

**Results:**

While statistically significant improvements in 5% to 83% of indicators were observed in each organization, more than 80% of indicators for breastfeeding, DDD and smoking cessation did not change. Statistically significant improvements were found in > 50% of indicators for asthma (52%), diabetes foot care (83%) and venous leg ulcers (60%). Organizations with > 50% improvements reported two unique implementation strategies which included hands-on skill practice sessions for nurses and the development of new patient education materials. Key facilitators for all organizations included education sessions as well as support from champions and managers while key barriers were lack of time, workload pressure and staff resistance.

**Conclusion:**

Implementation of nursing best practice guidelines can result in improved practice and patient outcomes across diverse settings yet many indicators remained unchanged. Mobilization of the nursing workforce to actively implement guidelines and to monitor the delivery of their care is important so that patients may learn about and receive recommended healthcare.

## Background

The gap between research evidence and clinical practice is one of the most persistent problems in the provision of quality health care. Approximately 30 to 40% of patients do not receive health care according to current scientific evidence and some patients receive unnecessary or harmful care [1,2]. In primary care, only 3% to 49% of patients receive recommended care according to a systematic review of studies of quality of care [3]. As one strategy to help health professionals and decision-makers address this gap, professional and government organizations are producing clinical guidelines with credibly appraised summaries of research results and recommendations for health care. The Guidelines International Network (GIN) was founded in 2002 and by 2004 there were 52 member organizations from 27 countries yet only two members were nursing organizations: The Joanna Briggs Institute for Evidence Based Nursing & Midwifery in Australia and the Royal College of Nursing Institute in the United Kingdom [4]. In the United States, The Agency for Healthcare Research and Quality (AHRQ) sponsors the National Guideline Clearinghouse which contains 105 nursing guidelines developed by 14 organizations in Canada, China, the United Kingdom and the United Sates [5].

One might question whether or not discipline-specific guidelines are required. We take the position that while health care is delivered to patients from a multidisciplinary perspective, the responsibilities of each team member need to be informed by the best available research evidence and some of this evidence is discipline-specific. The nursing profession represents a large proportion of health care providers and a potential workforce to address gaps in the provision of recommended health care.

The effectiveness of guideline implementation in nursing and allied health professions has received relatively little research attention. A Cochrane review summarized the results of 18 intervention studies of which 13 included physicians and nurses, and only 4 specifically targeted nurses [6]. The authors concluded that there is limited evidence supporting the effectiveness of guidelines for improved care in "professions allied to medicine" [6]. A systematic review of research evidence in nursing was conducted but only four studies met the inclusion criteria and the authors concluded that little is known about how to increase research use in nursing [7].

In contrast, there is a considerable body of physician-oriented and multi-disciplinary research with 235 studies reviewed by Grimshaw and colleagues (2004) of the effectiveness and costs of guideline development, dissemination and implementation strategies [8]. Physicians were the focus in 74% of these studies while the other studies targeted a multidisciplinary team. Results were promising in that eighty-six percent of studies reported improvements in care, however there were substantial variations in the results. Grimshaw and team concluded that further research is needed to support decisions about which guideline dissemination and implementation strategies are likely to be effective. No differences were found in effects according to the number of elements such as reminders, education, audit and feedback although Grimshaw et al. and others have suggested that using multifaceted strategies may be more effective than single interventions since they address multiple barriers to implementation [8-10]. While these systematic reviews help to advance our understanding of the potentially potent ingredients of guideline implementation programs, they summarize evidence from highly controlled research studies with targeted guidelines and pre-planned implementation settings. However, provincial government decision-makers who are determining whether or not to invest in nursing best practice guideline implementation programs, also need evaluation results from naturalistic implementation programs in diverse settings where decisions regarding which guidelines should be implemented and which recommendations from each guideline is a priority for implementation are determined by practitioners rather than by researchers.

In nursing, there are few empirical studies about the quality of care and it is crucial to examine the interrelationships between evidence-based nursing care processes and outcomes [11]. A state of the science review of nursing-sensitive outcomes describes a rapidly developing field yet many unresolved issues including what, when and how to measure indicators of nursing care [12]. Data about nursing practice is needed to answer questions about health human resource requirements and what nurses need to do to maximize their impact on the delivery of health care and patient outcomes.

In summary, there is a very limited evidence base for either the design of nursing guideline implementation interventions or for measuring the process and outcome of nursing care. An opportunity arose with the production of new nursing guidelines for a naturalistic study that examined implementation of various guidelines across settings to provide insights which are often absent from other studies where the protocol is tightly prescribed, only one guideline is implemented and it is implemented across homogeneous settings. The context for the study is described below.

### Context: The Registered Nurses' Association of Ontario Initiative

For the past seven years, the Registered Nurses' Association of Ontario (RNAO) in Canada has been leading the development and implementation of nursing best practice guidelines funded by the provincial government. Priority topics are selected using focus groups, surveys and an open web-based invitation to nurses. An expert panel is convened for each topic with representatives from different health care sectors (hospital, public health, long-term care), and responsibilities (administration, education, practice, research). Guidelines undergo extensive review by multidisciplinary stakeholders. Further details about the methodology for guideline development are described elsewhere [13,14]. To date, 30 guidelines have been published in English with twelve guidelines also translated into French [15]. Each guideline includes recommendations for practice such as patient assessment, patient education, medication monitoring, supportive care and referral to community services

We report on the real-time implementation of six guidelines during 2002–2004: asthma, breastfeeding, delirium-dementia-depression (DDD), foot complications in diabetes, smoking cessation and venous leg ulcers [15]. Specific objectives of this evaluation study were to document the process of best practice guideline implementation by topic, to describe facilitators and barriers to implementation and to determine the impact of indicators related to process and patient outcomes. The evaluation of these six guidelines with respect to patient referrals is reported elsewhere [16].

## Methods

A one-year prospective before and after implementation project was conducted with three months allotted for the baseline data collection, six months for implementation and three months for the follow-up data collection. The one-year time-frame was determined by the requirements of the government contract funding. The approach used was to evaluate guideline implementation as it naturally occurred in clinical settings. Ethics approval was received from the University of Ottawa Health and Social Sciences Ethics Review Board and from participating organizations. Following a call for proposals and a peer review process, eleven health care organisations were selected to implement one of the six guidelines. Organizations selected included four acute care teaching hospitals, two community hospitals, one chronic care hospital, one mental health teaching hospital, two home visiting nursing agencies and one regional public health unit. Sites began implementation of four topics in 2002 (asthma, breastfeeding, delirium-dementia-depression, and smoking cessation) and two topics in 2003 (foot complications in diabetes, and venous leg ulcers).

Each site committed in-kind resources including at least 10% of an administrator's time and paid educational time for nurses. Project funding supported the release of one full-time equivalent clinical resource nurse (CRN) for a year which included the implementation as well as the pre-implementation and post-implementation data collection. Some organizations elected to allocate this funding to more than one CRN as described in Table 1.

**Table 1:** Characteristics of organisations participating in the implementation of nursing guidelines

	**Best Practice Guidelines**					
**Organisational Characteristics**	**Asthma**	**Breastfeeding**	**Delirium, Dementia, Depression**	**Diabetes Foot Care**	**Smoking Cessation**	**Venous Leg Ulcers**

**Type and Number of Hospitals**	Community (1)	Teaching (1)	Teaching (3)	Community (1)	Teaching Mental Health (1)	Community Chronic Care (1)
**Units**	Emergency, Urgent care, Medicine	Labour & Delivery, Post-partum	Surgery, Medicine, Rehabilitation	Medicine	In-patients, clinics	Long-term care
**Community Nursing**		Regional Public Health Unit (1)		Home visiting (1)		Home visiting (1) Wound clinics (3)
**Number of Clinical Resource Nurses (CRN's)**	2 part-time	1 full-time	1 full-time manager as CRN, 7 APNs†	2 part-time	1 full-time manager as CRN, 7 APN's†	1 full-time
**Number of nurses educated**	200	68	211	179	67	65

† APN's or Advanced Practice Nurses

### Intervention

Initial workshops coordinated by the RNAO were held with three to five key stakeholders from each site (e.g. CRN, manager, administrator). Leaders from each guideline development panel gave structured presentations about the guideline recommendations with the supporting research evidence. A Toolkit [17,18] with a two-hour training session describing strategies for implementation of clinical practice guidelines was provided. The Toolkit was developed by an expert panel including members who have published on evidence-based nursing practice and theoretically-informed guideline implementation research [19-22]. The Toolkit was based on a model with six essential elements: guideline identification; stakeholder identification, assessment and engagement; environmental readiness; use of effective implementation strategies; evaluation of guideline implementation; and identification of resource requirements [18].

At each organization, a multidisciplinary steering committee reviewed the Toolkit and planned the implementation of the selected guideline in their setting. As recommended in the Toolkit, an environmental assessment of barriers and facilitators was undertaken, stakeholders were identified and specific implementation strategies were identified in a written action plan. Paid education sessions (range 2–9 hours) were provided by the CRNs for nurses and other health care providers. These sessions were interactive, incorporated problem solving, case studies and skill practice sessions. Table 2 describes implementation strategies including nurses' education, champions or local opinion leaders, reminder systems, policy review, creation of new documentation, multi-disciplinary involvement and patient education. In addition, monthly problem-solving teleconferences with all CRNs were held to discuss ongoing facilitators and barriers to guideline implementation. These teleconferences were led by experienced RNAO-based nursing consultants.

**Table 2:** Implementation strategies

**Bolded columns indicate guideline implementation that resulted in no change in > 80% of indicators**						
**Strategies**	**Asthma**	**Breastfeeding**	**Delirium, Dementia, Depression (DDD)**	**Diabetes Foot Care**	**Smoking Cessation**	**Venous Leg Ulcers (VLU)**

**Educational strategies for nurses**	Paid time 2 core sessions of 2 hours each for separate groups of nurses-inpatient and emergency Pre-learning package Articles about the project in internal newsletters Placebos to practice skills	**Paid time** **Core 4-hour training session off site** **Written material team teaching, non-didactic, focus on attitudes, beliefs and values, use of stories by mother/baby dyads** **Additional short in-services held**	**Paid time** **2–3.5 hours** **Powerpoint slides, facilitator guide, handouts, case studies, a game to review materials, standardized assessment tools**	Paid time Hospital: 1 session 30 to 60 minutes, as a lunch and learn, handouts, self-learning package Visiting nurses: 6 sessions for 1.5 hours each, practice sessions, word game, refresher training	**Paid time** **2 hours, Powerpoint slides, informal and interactive, stages of change theory** **Use of case scenarios depending on group e. g. in- patient, outpatient, long term care**	Paid time Manual (basic wound and VLU care) and CD for self-directed learning, individual 2-hour session with quiz, demonstration and bandaging practice by nurses Discussion of newsletters (not mailed) hospital nurses had demonstrations on bandaging and products
**Champions (Local opinion leaders)**	Encouraged stronger nurses to sign up early to be advocates and mentors		**Champions on each unit, part of the steering committee with role to raise issues in day to day rounds and to encourage the nurses to use the recommended assessment tools**		**A senior leader physician champion** **Several clinical resource nurses with one on each unit**	Resource people trained in both community and hospital settings Mentoring by consultants at client's home
**Reminder systems**	Logo, mugs, posters, name tags for nurses who completed the training		**Posters, binders, pocket cards listing symptoms of DDD**	Project logo, posters, articles in newsletters, voicemail messages, special flyer	**Buttons, posters, laminated pocket cards summarizing the steps of ask, advise, and assist strategy**	
**Policy Review**	yes	**Yes**	**Yes**	Yes	**Yes** **Smoking room policy changes**	yes
**Creation of new documentation**	Flow sheets Patient pathways	**Newborn critical pathways chart** **New charting tools and discharge sheets**	**Trigger questions added to initial pt assessment forms to help nurses maintain an index of suspicion**	New assessment tool		
**Multi-disciplinary involvement**	Respirologists very supportive but emergency physicians reluctant due to concern for nurses' workload	**"Little involvement of other disciplines"** **Conflict with dieticians in public health unit about 6 months excusive breast feeding**		Steering group formed with both hospital and community reps but did not have an active ongoing role	**Strong senior physician champion 'interdisciplinary work was amazing...the camaraderie between the disciplines and meeting everyone else from the different sites was one of the major benefits"**	Steering committee Community physician support
**Patient Education**	Patient education toolkits with placebos, teaching booklets and laminated cards on all units			Patient education and referral resources		Patient education brochure initiated but not completed

### Process and Patient Outcomes

In order to select key outcomes, a rapid three-way consultation occurred with the leader of the specific guideline development panel, decision-makers from each implementation site and a member of the research team. Criteria guiding final selection of outcomes included: outcomes with the strongest research evidence, outcomes feasible to measure in a short time frame, and outcomes relevant to priorities at participating organizations. Chart abstraction forms were adapted from tools used in previous research when available [23-27] and assessed for face validity and relevance by the decision-makers and research team.

Consecutive patient charts that met the inclusion/exclusion criteria were audited pre- and post-implementation. In addition, for one topic (asthma), patient techniques for use of medication inhaler device were observed [27]. In four out of six topics (asthma, breast-feeding, diabetes foot care, and smoking cessation), patients were interviewed in hospital or at home about recent nursing care and again, either four or eight weeks later by telephone, about their access to specialist and community resources [16]. For the topic of delirium-dementia-depression, participating units were concerned about ethical issues and the feasibility of obtaining informed proxy consent from family members within the three-month time frame for baseline data collection. The CRNs described a vulnerable population with many clients unable to give consent, substitute decision-makers difficult to access and some clients with no substitute decision makers. For the venous leg ulcers guideline, the home visiting nurse agency was willing to assist with the chart audit process but declined to participate in obtaining client interviews due to limited time and multiple obligations.

Patient eligibility criteria were defined for each guideline (Table 3). Our goal was to enrol 50 patients on each of the participating units in an eight week time frame. Response rates were calculated using the number of eligible patient charts as the denominator for the initial patient interview in hospital/home. The number of initial patient interviews was then used as the denominator for calculating the response rate for follow-up interviews since only those completing the first interview were eligible to be re-interviewed. Overall missing data were less than 10%.

**Table 3:** Best Practice Guideline Topics: Eligibility Criteria, Sources of Data, Response Rates

**Topic** Eligibility criteria	**Sources of Data**		
	
	**Chart Audit N**	**Patient Interview In Hospital – N, (% of chart audits)**	**Patient Telephone Interview at home N, % of 1^st ^Interview**
**Asthma** Adults with a previous diagnosis of asthma or are carrying a puffer.	Pre 80 Post 48 Decrease likely due to seasonal variations	Pre 31 38.8% Post 14, 29.2% Most seen in Emergency, few admitted to hospital	Pre 21, 67.7% Post 10, 71.4% 4 weeks after discharge
**Breastfeeding** Singleton infant, term pregnancy, no major abnormalities, not up for adoption, discharged home with mother	Pre 103 Post 89	Pre 75, 72.8% Post 80, 89.9%	Pre 51, 68.0% Post 61, 76.3% 8–9 weeks after birth
**Delirium, Dementia Depression** Include if ≥ 65 years	Pre 196 Post 187	N/A	N/A
**Diabetes Foot Care** Adults with Type 1 or Type 2 Diabetes excluding women with gestational diabetes	Pre 98 Post 123	Pre 66, 67.3% Post 58, 47.2%	Pre 38, 57.6% Post 26, 44.8% 4 to 6 weeks after discharge
**Smoking Cessation** 18 years or older, daily or occasional smoker in mental health facility Excluded if major depression or psychiatric illness	Pre 116 Post 93	Pre 89, 76.7% Post 84, 90.3%	Pre 42, 47.2% Post 27, 32.1% 4 weeks after admission
**Venous Leg ulcers** New or recurrent, Exclude if arterial ulcer, vasculitis, under diabetic management	Pre 109 Post 52	N/A	N/A

Pre: Pre-implementation; Post: Post-implementation

The data collection process was supervised centrally by an experienced Project Manager who frequently communicated with the CRNs to maintain the quality of data collection. The CRNs extracted pilot data from five to 10 charts at each organization to develop an effective system for obtaining charts and to assess the feasibility of coding categories. Part-time data collectors were then trained and supervised by the CRN locally to conduct the chart audits. The Project Manager reviewed data from all sites on an ongoing basis and contacted local CRNs about missing data or inconsistencies.

Descriptive statistics and bivariate statistics (independent t-tests, Pearson chi-square) were used to describe characteristics of the sample, and to compare before and after measures of clinical outcomes within study sites. Fisher's exact test was used when expected frequencies were small.

### Qualitative Interviews

Semi-structured interview schedules were developed by the co-investigators and administered to all CRNs, administrators and selected staff nurses immediately after the implementation. The staff nurses (2–3 per unit) were identified by the CRN to include those who had a favourable as well as those who had a less favourable experience with the guideline implementation.

Questions were designed to elicit information about the strategies used as well as the perceived factors that facilitated or provided barriers to guideline implementation. In addition, CRNs were interviewed at the mid-point of implementation to describe priority recommendations selected for implementation and reasons why some recommendations were not selected. Examples of these interview schedules as well as our approach used to conduct and analyze interview data are provided in an available monograph [28].

The Project Manager and trained interviewers conducted the interviews which were audio-taped with the consent of each respondent. Analysis of the interviews was conducted using QSR-N5. Interviews were initially coded by one individual using a coding guide. Content analysis was conducted looking for prominent themes and patterns across respondents for each guideline [29].

## Results

### Chart audits and patient interviews

Completion rates for chart audits were greater than 95% for all eligible cases. Response rates varied for initial and follow-up patient interviews, as displayed in Table 3, with an average of 61.1%. Language barriers and refusals lowered the response rates at the initial interviews. For the follow-up interviews, the response rates decreased due to drop-outs, difficulty contacting patients after discharge, discharge of patients to other facilities and death. In addition, for the asthma guideline, lower response rates were due to an extremely busy emergency department discharging potential patients before they could be interviewed for the study.

As described in Table 4, there were statistically significant improvements across all topics with improvements in more than half of the indicators for asthma (52.4%), diabetes foot care (82.6%) and venous leg ulcers (60.0%). Table 5 displays the details of selected outcomes pre- and post-guideline implementation according to chart audit data.

**Table 4:** Description of recommendations and proportion of indicators which improved or not from pre to post implementation

	**Asthma**	**Breastfeeding**	**Delirium, Dementia, Depression**	**Foot Complications Diabetes**	**Smoking Cessation**	**Venous Leg Ulcers**
**Recommendations implemented**	4//4	3/7	7/7	6/6	3/7	52/56
**Content of recommendations implemented†**	-Assess asthma control -Educate re Asthma -Develop action plans -Assess inhaler device technique	-Endorse WHO Baby Friendly Hospital Initiative Advocate friendly environments -Promote community action -Assess Postnatal factors -Educate and support	-Maintain suspicion for DDD in the older adult -Screen for cognition, function, behaviour and mood -Use structured assessment & standardized tools	-Physical exam of feet to assess risk factors -Educate about foot care Educate tailored to knowledge, needs and risk factors	Minimal smoking cessation intervention using ask, advice, assist, arrange protocol Recognize relapse is common and need to re-engage.	-Assess risk factors -Measure surface areas -Ankle Brachial Pressure Index -Assess Pain Debride wound -Assess Infection -Graduated compression bandaging
**Number of chart audit items**	12	4	19	13	12	15
Improvements*	7	0	3	12	4	9
No difference	5	3	16	1	8	6
Worse*	0	1	0	0	0	0
**Number of patient interview items**	5	9	N/A	7	12	N/A
Improvements*	1	0		7	0	
No difference	4	9		0	12	
Worse*	0	0		0	0	
**Observation**	1	N/A	N/A	N/A	N/A	N/A
Improvements* (Inhaler use)	1					
**Number of items in follow-up telephone call**	3	6	N/A	3	11	N/A
Improvements*	2	1		0	0	
No difference	0	5		3	10	
Worse*	0	0		0	1	
**Total # indicators **N%	21	19	19	23	35	15
Improvements*	11 52.4%	1 5.3%	3 15.8%	19 82.6%	4 11.4%	9 60.0%
No difference	10 47.6%	17 89.5%	16 84.2%	4 17.4%	30 85.7%	6 40.0%
Worse*	0	1 5.3%	0	0	1 2.9%	0

* p < 0.05, N/A or not applicable, †Excludes recommendations about referrals which are discussed in another paper [ref referrals paper] For full description see [16]

**Table 5:** Selected clinical outcomes at pre and post guideline implementation ‡

**Guideline**	**Selected process of care indicators and patient outcomes**	**Pre %**	**Post %**	**p-value**	**Changes† Source of Data**
**Asthma** N = 80 Pre N = 48 Post	Assessment of patient use of β_2 _agonist	52.5	72.9	0.026	+ chart
	Individual action plan for client discharge	3.8	23.9	0.001	+ chart
	Teaching information provided	3.8	27.1	0.000	+ chart
**Breastfeeding**	Breast-feeding in hospital N = 75 Pre; N = 76 Post				- Interview in hospital
	Exclusive	80.0	67.1		
	Mixed	20.0	22.4	0.012	
	Formula only	0.0	10.5		
	Infant supplementation given N = 103 Pre; N = 89 Post	24.3	34.8	0.115	ns chart
**Delirium, Dementia and Depression** N = 196 Pre N = 186 Post	Assessment on admission for memory problems	66.3	76.9	0.024	+ chart
	Assessment on admission for mood (i.e. depression)	29.6	45.0	0.003	+ chart
	Assessment during stay for memory problems	62.4	72.7	0.037	+ chart
**Diabetes Foot Care** N = 97 Pre N = 119 Post	Assessment for risk factors for foot ulceration/amputation	16.5	60.7	0.000	+ chart
	Monofilament used to assess sensation in the feet				+ chart
	Yes	0.0	57.1	0.000	
	No	75.8	5.9		
	No note	24.2	37.0		
**Smoking Cessation**	Received advice on stopping smoking or staying quit N = 116 Pre; N = 93 Post	1.7	17.4	0.000	+ chart
	Self-help information given N = 116 Pre; N = 93 Post	1.7	28.0	0.000	+ chart
	Have you tried to quit smoking in the past 2 months? N = 36 Pre; N = 21 Post	66.7	33.3	0.026	- Telephone call at home
**Venous Leg Ulcers** N = 87 Pre N = 52 Post	Assessed for clinical history and features associated with venous disease	58.6	96.2	0.000	+ Chart
	Doppler measurement of Ankle Brachial Pressure Index (ABPI)	3.7	45.7	0.000	+ Chart

† According to recommendation + indicates improvement with p < 0.05, – indicates worse care with p < 0.05, ns indicates no significant difference in care

**‡ **Selection illustrates nursing care and patient outcomes from chart audit data as well as the only two statistically significant negative changes (-).

Overall, with respect to chart audit data, there were statistically significant improvements in nearly half of the indicators (46.7%, 35/75). It is interesting to note that improvements were found in items related to: patient assessment/risk classification 37%; patient education 29%; medication and treatments 14%; symptom monitoring 9%; supportive care 6%; and other 6%. Statistically significant improvements were also found in pre- and post-observation scores of asthma patients' correct use of an inhaler device (p =.014). With respect to patient interview data, it is notable that statistically significant improvements were found in all seven indicators for diabetes foot care as reported in Table 4.

No statistically significant differences were found > 80% of the indicators for three guidelines (breastfeeding, DDD, smoking cessation). In addition, two statistically significant negative results were found for two of these guidelines. Specifically, fewer mothers were exclusively breastfeeding their infants in hospital (80.0% pre-implementation, 67.1% post-implementation; p = 0.012) and fewer mental health patients reported that they had tried to quit smoking post-guideline implementation (66.7% pre-implementation, 33.3% post-implementation; p = .026).

The concordance of nursing practices with indicators measured varied considerably post-implementation. The lowest level was 17.4% for the provision of smoking cessation advice to smokers in a mental health organization and the highest level was 96.2% for the patients in the chronic care hospital and the clients receiving community-based nursing services for the care of their venous leg ulcers (Table 5).

Even in the three guidelines with statistically significant improvements in > 50% of indicators, there were still substantial gaps in the provision of evidence-based recommendations at post-intervention as follows: Only 23.9% of hospital patients with asthma had an action plan at discharge; only 60.5% of hospital and community-based patients with diabetes had a documented assessment of risk factors for foot ulcers; and only 45.7% of patients with venous leg ulcers from long-term care and a community-based nursing service received a Doppler Assessment of Ankle BrachialPressure Index (Table 5).

### Qualitative interviews

Participation in the qualitative interviews by the CRNs, nurses and administrators was strong from all units with an overall participation rate of 87.4% (83/95) with a range of 75%–100% per guideline. The proportion of recommendations implemented varied by guideline topic from 43% (e.g. 3/7) to 100% (e.g. 7/7). A very brief synopsis of the content of recommendations is described in Table 4 with a full description of recommendations available [15]. For three guidelines, the CRNs reported at mid-point that their implementation action plan addressed all clinical recommendations (asthma, DDD and diabetes foot care). However, for the other three topics the CRNs explained reasons why certain recommendations were not a priority as described below.

#### Breastfeeding

The prenatal components which included a comprehensive assessment to facilitate a breastfeeding plan and small informal group education classes were not implemented because "*these are very expensive and would require too much personnel time and it is felt not possible at this time*". Also, the recommendation to conduct an in-person follow-up and assessment if a mother and baby were discharged within 48 hours of birth was not implemented due to perceived lack of available public health nurses. Controversy arose about the recommendation for exclusive breastfeeding for the first six months of life from the hospital nutrition/dietetic staff. According to the CRN, these nutritionists followed other national guidelines recommending exclusive breastfeeding for only four months.

#### Smoking Cessation

The CRN reported that three recommendations were not implemented: provide intensive smoking cessation interventions, implement interventions with pregnant women and encourage smokers and non-smokers to have smoke-free homes. Reasons for non-implementation included lack of time for nurses to do intensive smoking cessation counselling and the availability of experts at a nicotine dependence clinic within the organization. The pregnant women population was not applicable to the participating mental health organization. With respect to the quest for smoke-free homes of patients/clients, the CRN stated *"We felt that it was something that we did not have a lot of control over, some clients live in rooming houses and boarding houses and collective group settings."*

#### Venous Leg Ulcers

Of the 56 recommendations, 52 were implemented. Recommendations not implemented related to secondary treatment options including venous surgery, electrical stimulation, hyperbaric oxygen, and therapeutic ultrasound. The CRN explained that "*these are second wave treatment option, so the first part of our process has really been trying to educate the nurses around the guidelines, care and so that sort of comes after the initial care."*

### Overall Facilitators

Participants' perceptions (CRNs, managers, staff nurses) of the most important facilitators were remarkably similar across implementation sites, with the most common including the education sessions, support from champions such as the CRN, APNs, and physicians, organizational and administrative support (e.g., staff replacement time to attend educational sessions), and involvement of multiple stakeholders (Table 6). Participants agreed that the following also facilitated BPG implementation: team work and collaboration, RNAO support, specific resources such as Asthma Education Centre, and availability of supplies (e.g., wound compression supplies, Doppler ultrasounds).

**Table 6:** Participants' perceptions of most important facilitators for implementation

**Asthma**	**Breastfeeding**	**Delirium, Dementia, Depression**	**Diabetes Foot Care**	**Smoking Cessation**	**Venous Leg Ulcers**
Education sessions	Education sessions	Education sessions	Education sessions	Education sessions	Education sessions
Administrative support and buy-in	Nurses' paid time for attending education sessions	Support of CRNs‡, APNs† on units	Ease of implementation, easy to use tools	Organizational support and readiness	Buy-in of nursing staff
Support of CRN‡	Support of CRN‡	Steering committee with representation from all units	Support of CRN‡	Champions including physician	Support of CRNs‡ and other educators
Having education as mandatory program outside of work hours	Partnership between hospital and public health settings	Organizational and management support	Organizational commitment and management support	Management support	Organizational support and resources
Having credible person in department to facilitate implementation	Rooming-in	Having all stakeholders on board, managers, APNs†, nurses, champions	Involving staff at start of process	Ongoing access to information	Increased number of VLU patients

† APNs or Advanced Practice Nurses

‡ CRN or Clinical Resource Nurse

### Overall Barriers

Participant perceptions of the most important barriers to implementation included some common barriers across all topics and sites such as workload and time pressures, competing demands, staff resistance and lack of buy-in, lack of support from other organizational levels, and organizational change. Other barriers were specific to the implementation sites, such as short patient stays, lack of support from physicians, and lack of changed documentation (Table 7). A need for the ongoing analysis of stakeholders' views and barriers was suggested by the CRN *"Although considerable time was spent conducting a stakeholder analysis, the committee recognized that as changes occurred, more work was required in analyzing, revising and engaging stakeholders."*

**Table 7:** Participants' perceptions of most important barriers to implementation

**Asthma**	**Breastfeeding**	**Delirium, Dementia, Depression**	**Diabetes Foot Care**	**Smoking Cessation**	**Venous Leg Ulcers**
Lack of time to work with patients in emergency department	Staff resistance	Workload and competing demands	Time and workload pressure for nurses	Client resistance to smoking cessation	Time and workload pressures
Too few asthma patients on in-patient units (not peak asthma season)	Public Health Nurses' limited access to CRN and lactation consultant	Limited time spent with patient, patient stay too short	Difficulty getting support and buy- in from all levels of organization (managers, nurses, physicians)	Time and workload pressures, and competing demands	SARS outbreak created delay in education and implementation
Timing of project, timing of launch, lost momentum	Workload and limited availability of CRN‡in hospital	Complexity of skills required for RPNs§	Patient issues: cost of taking action, patient motivation, communication, follow-up	Challenges of administration and coordination across four sites	Lack of physicians willingness to order high compression bandaging
Change in management in two key units	Lack of communication between hospital and public health	Lack of buy-in from nurse managers at unit level and some nurses	Lack of CRN‡ for a period of time, delay in appointing new CRN	Attitudes re clients, past experience led to belief clients can't quit smoking	Format of education manual
Lack of physician and administrative support in emergency; physicians in a hurry to send patients home	Lack of management support	Changes in senior personnel and lack of consistent champion	Organizational change, reorganization	Documentation not changed for staff to record assessment of smoking cessation	Lack of educational material for clients

† APNs or Advanced Practice Nurses

‡ CRN or Clinical Resource Nurse

§ RPN or Registered Practical Nurses

## Discussion

After the one-year implementation of newly developed nursing-oriented guidelines, a substantial proportion of patients were not receiving recommended nursing care. While our results reveal that some statistically significant positive improvements were found for all guideline topics, more than 80% of the indicators for breastfeeding, DDD and smoking cessation did not change from pre-implementation to post-implementation. However, there were statistically significant improvements in more than half of the indicators for the asthma, diabetes foot care and venous leg ulcer guidelines. These improvements are notable given the relatively short time frame of one-year for project completion.

Overall, this situation is consistent with findings of other studies of guideline implementation and assessment of the quality of care [1-3,6,8]. Perhaps changes in the processes of care and outcomes would have been enhanced if nurses, managers and other disciplines were aware of the small proportion of patients actually receiving guideline-based recommended care. In this study, feedback on the pre-intervention study results was not provided to the agency or staff. Comparative data of nursing practice and patient outcomes is important for health service delivery to determine the quality and quantity of care provided. Systematic reviews of audit and feedback conclude that telling people what they have been doing does impact on change and improve professional practice [30].

Recent studies of nursing practice including subsequent work of our own suggest that it is possible to obtain data in a reliable and valid way [31-33]. Electronic gathering and dissemination systems that document patients' responses to treatment, obtain real-time outcome feedback and support access to electronic resources including RNAO guidelines using hand-held computers at point of care are promising tools to support guideline implementation [34].

The implementation strategies used in this study reflect those generally classified as multi-faceted interventions by Grimshaw and colleagues [8]. We observed two unique strategies applied to each of the three guideline implementations resulting in improvements in more than 50% of the indicators. Firstly, these nurses' received an opportunity for hands-on skill practice sessions (trial use of placebo devices for asthma medication, practice sessions for foot assessment of people with diabetes, and practice sessions for bandaging for people with venous leg ulcers). Skill practice has been identified as an element of interactive workshops which may also include role-play or case discussion. Interactive workshops can result in moderately large changes in professional practice compared to didactic lecture-only sessions which are unlikely to change behaviour [35]. However, it is noteworthy that case study discussions were used in education sessions for the three other guidelines without subsequent changes in professional practice (breastfeeding, DDD and smoking cessation). The skill practice component appears to be a vital element and warrants attention.

The second unique implementation element used with the three guidelines with substantial practice changes was the development of patient education toolkits and brochures. The CRN for the asthma guideline suggested that the new patient information tool provided an opportunity to reinforce previous teaching provided to nurses. The CRN for diabetes foot care reported that patients appreciated the education suggesting a positive feedback process from clients may have encouraged implementation of the guideline. It is noteworthy that all seven process and outcome indicators from the patient interviews increased significantly from pre to post-implementation for the topic of diabetes foot care.

For other guideline topics, positive feedback from patient education was less likely in part due to the nature of the guideline topic and in part due to the application in the selected clinical context. While the diabetes guideline was directly applicable to the day-to-day care in the medical hospital in-patient units and the ongoing care of home visiting nurses, the smoking cessation guideline required a considerable shift in the norms at the participating mental health facility where previously, cigarettes had been provided as a positive reward for good patient behaviour. With respect to the breastfeeding guideline, the post-partum nurses did not receive feedback about the impact of their hospital-based teaching because public health nurses assumed care once the mother and infant were discharged from the hospital. The public health nurses did not receive feedback about long-term breast-feeding outcomes because their teaching was concentrated in the early post-partum period. The uptake of guidelines such as breastfeeding or smoking cessation with a preventive focus appears to be slower, a finding that is consistent with Rogers contention that preventive innovations have a particularly slow rate of adoption because individuals have difficulties perceiving the relative advantage and the consequences are distant in the future [36].

Management support was reported as an important facilitator for all guideline implementations except for breastfeeding. We report in this study and in previous evaluations of RNAO guidelines [37], that one of the most important facilitators for implementation of a guideline is management support and commitment. In addition, key barriers documented in previous work and in this report include lack of administrative support and changes in management. We note that "leadership support for evidence-based practice culture" is an explicit organizational element of Stetler's conceptual framework [38] and that leadership is a sub-element of "context" of the Promoting Action on Research Implementation in Health Services (PARIHS) framework [39]. However, management support and leadership are not listed as an implementation strategy in either our implementation Toolkit [17,18] or with the current EPOC Cochrane systematic reviews of consumer, professional or organizational change. We conceptualise "managerial leadership for research use" as a multidimensional process of influence to enable nurses to use research evidence in clinical practice [40,41]. An integrative review of 12 published studies found that managerial support, policy revisions, auditing, role modelling and valuing research facilitated nurses' use of research evidence [41]. We suggest that managerial leadership is an important element for guideline implementation in nursing

With respect to process and patient outcomes, we found that none of the organizations had existing patient care databases with sufficient detail or data about nursing process of care to be used in the evaluation of these RNAO guidelines. Thus, we endeavoured to rapidly adapt existing published tools and develop new tools (chart audit, observation and interview questionnaires) to measure the processes of nursing care that are meaningful and actionable by nursing managers and senior administrators. These data collection tools were assessed for content validity and applicability and are available on the web with the RNAO Cycle 3 guidelines [15] and may provide a launching point for other teams evaluating gaps in nursing care. Subsequent psychometric testing of the asthma observation tool found mean inter-rater reliability indicies of .82 [27]. Other related articles, monographs and user-guides describing measures for evaluating the implementation of nursing best practice guidelines are available [27,28,33,42,43].

An environmental scan of facilitators and barriers as recommended in the implementation Toolkit [17,18] was conducted by the CRNs for each guideline implementation in order to plan the intervention strategies in consultation with a multidisciplinary steering committee. However, knowing that a barrier(s) exists is only the first step. There are no quick fixes for many of the barriers identified by CRNs. For example, overcoming nurses' resistance to the breastfeeding guideline recommendations, addressing competing demands for time as reported for the DDD guideline and tackling long-standing issues of client resistance to smoking cessation are all complex issues. Tailored interventions to overcome identified barriers to change have been evaluated in a review of 15 studies with mixed results because it was not clear whether all barriers or important barriers were identified and addressed by pre-selected strategies [44]. Pragmatic evaluations of guideline implementation in nursing are necessary to document important barriers for the design and evaluation of tailored strategies that fit real-world implementation.

A note of caution is required about the evaluation of recommendations that are sensitive to patient preferences and nurses' values. As one report advises [45], performance measures require attention to avoid defining high testing rates as good quality of care, since they may not take into consideration patient preferences for care options. Patient preferences and nursing values and beliefs are reported to influence evidence-based nursing [19] and may have contributed to the two statistically significant negative outcomes found in this study. With respect to breastfeeding, the CRN reported that tension existed about the extent to which staff should encourage individual mothers to breastfeed versus elicit mothers' choices to bottle-feed their infant. With respect to smoking cessation, client resistance to smoking cessation was reported as an important barrier by nurses. Evidence-based practice is defined to "integrate the best research evidence with clinical expertise and patient values [46]." There is a need to identify patient and provider values implicit in guideline recommendations and these values should be reported along with the research evidence for the recommendations [47].

Lack of time and workload considerations were reported as barriers across all guideline implementation initiatives. Cost considerations such as staffing for increased workload due to new procedures recommended in guidelines are critical for decision-makers [8], yet few authors have attempted to estimate these costs. In the subsequent Cycle 4 evaluation projects, cost estimates were derived for implementation of the pressure ulcer guideline and this report is available on request from the authors [48]. The cost of implementation during the initial six month start-up phase and in the subsequent 18 months were determined using a balanced score card approach. Education and capital costs were high in the initial period, while patient operating costs accounted for the largest set of expenditures in the latter period. Future studies are needed to compare the cost implications of guideline implementation across settings and among different types of guidelines

### Study Limitations

Organizations in this evaluation study volunteered to participate and are therefore a self-selected group. Their volunteer status may have stimulated guideline recommendation uptake and we note that these organizations may reflect the early adopters and innovation champions [36]. Indeed the CRNs in our study appeared to possess characteristics of early adopters with well developed interpersonal and negotiating skills, often holding key linking positions in their organization. Other limitations include the lack of concurrent control groups and the lack of inter-rater reliability testing of chart auditors. Thus, we cannot be certain that the observed changes would have happened without the intervention. Future studies with concurrent control groups and thorough process evaluations are recommended. Periodic feedback to CRNs, managers and staff nurses regarding the status of implementation, assessment of ongoing barriers and the design of repeated opportunities to infuse this information in a tailored implementation plan are suggested for future research.

### Study Strengths

A unique opportunity to study the process and impact of six newly developed nursing guidelines in hospital and community settings existed. We report a real-world implementation project that provides data about promising implementation strategies.

Specifically, these strategies include the opportunity to practice skills in interactive educational sessions for nurses, the development and use of patient educations tools, and the importance of managerial leadership for nurses' use of research-based guidelines. The lessons learned in this evaluation project may be helpful in planning future guideline implementations and in planning future research.

With funding from the Canadian Health Services Research Foundation, the Canadian Institutes of Health Research and the Canadian Nurses Foundation, we are currently conducting follow-up studies to examine the long-term impact of nursing guideline implementation on clinical and process outcomes. This follows from the comments of CRNs and managers who observed that it takes longer than the six months allocated to the intervention to make clinical changes. In addition, our longitudinal study examines the predictors of sustained implementation of guideline recommendations [49].

In Canada, there is a strong public and political agenda to continuously strive towards high quality health care. The 10-year action plan to strengthen health care includes a priority to share best practices and provide information to make progress transparent to citizens [50]. The nursing profession, a major group of healthcare providers, needs to share intervention strategies and evaluation results to improve health care delivery. Developing, implementing and evaluating best practice guidelines in *nursin*g are essential elements in high quality health care.

## Conclusion

Clinical practice guidelines have the potential to improve the quality of care received by patients and thereby improve patient outcomes. Yet there is a lack of evidence about the impact of *nursing *best practice guidelines and the most effective strategies to implement such guidelines. We found that a multi-faceted intervention for the implementation of *nursing *guidelines about asthma, breastfeeding, delirium-dementia-depression, reducing foot complications in diabetes, smoking cessation and venous leg ulcers can result in some improvement in practice and patient outcomes across diverse healthcare settings yet many outcomes remained unchanged. Promising elements for future initiatives are identified including interactive education with skill practice sessions, attention to patient education, involvement of managerial leadership and electronic gathering and dissemination systems offering both real-time feedback and access to guidelines.

## Competing interests

All authors declare that they have no competing interests. Tazim Virani was contracted by the RNAO to lead the overall initiative and work in collaboration with the other authors in pilot testing and evaluating the best practice guidelines.

## Authors' contributions

BD and NE were Co-Principal Investigators and conceived and designed the evaluation of this project. TV led the implementation strategies, reviewed the action plans of the Clinical Resource Nurses and was involved in providing input in draft measures and protocols. BD, NE, JP designed data collection measures and protocols. All authors contributed to the interpretation of data and the preparation of this manuscript.

## Funding

We acknowledge project funding from the Government of Ontario. Dr. Edwards holds a Nursing Chair funded by the Canadian Health Services Research Foundation, the Canadian Institutes of Health Research and the Government of Ontario. Dr. Davies held a Career Scientist award funded by the Ontario Ministry of Health and Long-Term Care at the time of this study. Dr. Ploeg held an Investigator Award from the Canadian Institutes of Health Research and St. Joseph's Health Care, Hamilton, Ontario. Tazim Virani holds a doctoral fellowship funded by the Ontario Ministry of Health and Long-term Care and awarded by the RNAO. The views expressed in this publication are those of the authors and not those of the RNAO or the Government of Ontario.

## Pre-publication history

The pre-publication history for this paper can be accessed here:


